# Fifteen-Year Population Attributable Fractions and Causal Pies of Risk Factors for Newly Developed Hepatocellular Carcinomas in 11,801 Men in Taiwan

**DOI:** 10.1371/journal.pone.0034779

**Published:** 2012-04-10

**Authors:** Shu-Fen Liao, Hwai-I Yang, Mei-Hsuan Lee, Chien-Jen Chen, Wen-Chung Lee

**Affiliations:** 1 Research Center for Genes, Environment and Human Health, and Institute of Epidemiology and Preventive Medicine, College of Public Health, National Taiwan University, Taipei, Taiwan; 2 Molecular and Genomic Epidemiology Center, China Medical University Hospital and Graduate Institute of Clinical Medical Science, China Medical University, Taichung, Taiwan; 3 Genomics Research Center, Academia Sinica, Taipei, Taiwan; University of Ottawa, Canada

## Abstract

Development of hepatocellular carcinoma (HCC) is a multi-factorial process. Chronic infections with hepatitis B virus (HBV) and hepatitis C virus (HCV) are important risk factors of HCC. Host factors, such as alcohol drinking, may also play a role. This study aims to provide a synthesis view on the development of HCC by examining multiple risk factors jointly and collectively. Causal-pie modeling technique was applied to analyze a cohort of 11,801 male residents (followed up for 15 years) in Taiwan, during which a total of 298 incident HCC cases were ascertained. The rate ratios adjusted by age were further modeled by an additive Poisson regression. Population attributable fractions (PAFs) and causal-pie weights (CPWs) were calculated. A PAF indicates the magnitude of case-load reduction under a particular intervention scenario, whereas a CPW for a particular class of causal pies represents the proportion of HCC cases attributable to that class. Using PAF we observed a chance to reduce around 60% HCC risk moving from no HBV-related intervention to the total elimination of the virus. An additional ∼15% (or ∼5%) reduction can be expected, if the HBV-related intervention is coupled with an HCV-related intervention (or an anti-drinking campaign). Eight classes of causal pies were found to be significant, including four dose-response classes of HBV (total CPW=52.7%), one independent-effect class of HCV (CPW=14.4%), one HBV-alcohol interaction class (CPW=4.2%), one HBV-HCV interaction class (CPW=1.7%), and one all-unknown class (CPW=27.0%). Causal-pie modeling for HCC helps clarify the relative importance of each viral and host factor, as well as their interactions.

## Introduction

Hepatocellular carcinoma (HCC) is one of the most common cancers in the world [1]. It represents 7.9% in men and 6.5% in women, of the total new cancer cases reported each year [1]. Worldwide, the incidence rate is 10.8 per 100,000 person years (16.0 for males and 6.0 for females) [1]. Taiwan is considered a high incidence country, and the incidence rates reported from national cancer registry in 2007 are 52.8 and 20.5 per 100,000 person years in men and in women, respectively [2].

Chronic infection with hepatitis B virus (HBV) has been recognized as a major cause of HCC [3]–[8]. More than 350 million persons live with HBV infection worldwide [9], [10]. Previous epidemiologic studies showed that the risk of HCC associated with HBV infection ranges from 5-fold to 98-fold with a population attributable fraction (PAF) of 8% to 94% [3]. A significant dose-response relationship was also observed between HBV DNA level (which quantifies viral replication in human body) and HCC risk [9], [11], [12]. Hepatitis C virus (HCV), with 170 million persons all over the world infected with it, is another important cause of HCC [3], [5], [8], [13], [14]. Results from a meta-analysis show that there is a remarkable geographic variation for the association between HCV and HCC risk, with the odds ratio ranging from 11.5 in countries at high HBV endemicity such as Taiwan and sub-Saharan Africa to 31.2 in countries predominant for HCV infection such as Japan [15]. A dose-response relationship can also be demonstrated between HCV RNA level and HCC risk [13].

Although the independent effects of the above HBV and HCV infections had been well characterized, their possible ‘interactions’ are less known (whether co-infection of HBV and HCV increases or decreases the predicted HCC risk calculated from a simple multiplication or addition of the two risks corresponding to each individual virus, and by how much?) [3], [14], [16]–[18]. Also, development of HCC is a multi-factorial process [3], [19]. Besides viral factors, ‘host’ factors (such as alcohol drinking and cigarette smoking) may also play certain roles [3]. They may also exhibit some sorts of interactions, including the host-host type or the host-virus type [20]–[22]. In this study, we apply a newly developed causal-pie modeling technique [23] to analyze a cohort conducted in Taiwan—the community-based cancer screening program (CBCSP) [16]. We aim to provide a synthesis view on the development of HCC by examining multiple risk factors jointly and collectively. We also quantify the relative importance of each viral and host factor, and their interactions.

## Materials and Methods

### Recruitment and Follow-up of Subjects

The CBCSP cohort started in 1991 to 1992 [16]. A total of 89,293 individuals aged 30 to 65 years residing in seven townships (Sanchi, Chutung, Potzu, Kaoshu, Makung, Paihsa, and Huhsi) in Taiwan were invited. A total of 23,820 individuals agreed to participate and provided the written informed consents for interview, health examination and blood collection. Demographic data for residents who did not participate in the study were quite similar to those of residents who agreed to participate except in educational level [24]. Standardized personal interviews were conducted to obtain baseline information on socio-demographic characteristics, cigarette smoking, alcohol drinking, etc. At study entry, all participants received abdominal ultrasonography and donated their blood for various serological tests including hepatitis B surface antigen (HBsAg) and antibodies against hepatitis C virus (anti-HCV). Further quantification of HBV DNA or HCV RNA loads was done for those participants with seropositivity of HBsAg or anti-HCV [9], [13]. The CBCSP cohort participants were followed up until December 31, 2006. During the follow-up period, a total of 437 newly developed HCC cases (298 for men and 139 for women) were ascertained through the computerized data linkage with national cancer registry in Taiwan. The ascertainment was ensured to be complete and accurate by the verification with the profiles on the national death certification system.

### Restriction on Male Subjects

In this study, we restricted our analysis on the male subset of the cohort to specifically investigate causal pies of risk factors for HCC in men. (Pathogenesis of HCC has been considered different between men and women [3], [25], [26]. Sexual comparison of HCC causal pies should be an interesting topic for further study.) The distributions of township residence, age and education among male responders and non-responders were presented in Table S1. Overall, men who participated in the cohort are older, and with a higher-proportion having low-level education, than those who did not. Some differences in residential area distributions between responders and non-responders were also noted.

A total of 11,801 men who were free of liver cirrhosis and HCC at study entry were included for the present study. The study was approved by the Institutional Review Board of the College of Public Health, National Taiwan University (Taipei, Taiwan).

### Causal-Pie Modeling

Liao and Lee's causal-pie modeling technique [23] was applied to analyze the data. It consists of the following four steps: 1) adjusting for confounders (age in this study) using an ordinary (multiplicative) model, 2) building an additive model with non-negative parameters using a stepwise model selection algorithm, 3) calculating population attributable fractions (PAFs) to present the fraction of case subjects that would be prevented under various public health intervention or treatment strategies, based on the final model built in the previous step, and 4) solving a system of PAF equations to obtain the causal-pie weights (CPWs; defined as proportion of case subjects attributable to a particular class of causal pies). It should be noted that the additive model in step 2 also uses the ratio-scale indices, such as an odds ratio (OR) or a rate ratio (RR), to gauge exposure-disease relations. ‘Additive’ here indicates that the ORs or RRs themselves follow a ‘linear’ relation (instead of a ‘log-linear’ relation) and not that we are resorting to difference-scale indices, such as a risk difference or a rate difference.

In addition, we extend the method of Liao and Lee [23] in two ways to encompass the scenarios in this study. First, the present study is a cohort study with person-time data instead of a case-control study with pure-count data considered in Liao and Lee's paper [23]. Therefore, we change the fundamental index from an OR (for pure-count data) to an RR (for person-time data) and the regression method from a ‘logistic regression’ (for pure-count data) to a ‘Poisson regression’ (for person-time data). Second, Liao and Lee [23] only considered risk factors that are binary (exposed vs. non-exposed), whereas, in this study the risk factors can be in binary and ordinal scales. For a binary factor, we can use a single variable to represent it: coded ‘1’ when exposed, ‘0’ otherwise. For an ordinal factor with a total of 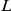 levels (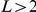), we resort to ‘incremental codes’. To be precise, let the ordinal factor be denoted as 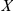, with 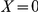 representing its lowest (or unexposed) level and 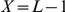, its highest exposure level, the incremental codes for 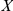 are (a total of 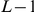 codes for an 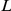-level factor):
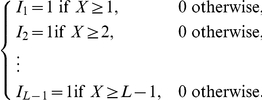
With the 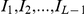 so defined, the regressions are proceeded using these artificially created incremental codes as the regressors. (Note that the interaction terms between the incremental codes of the same risk factor are not allowed.) And then the calculations of PAFs and CPWs are straightforward as described in Liao and Lee [23].

All the analyses were performed using SAS version 9.1. The significance level was set at 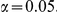 The additive Poisson regression was fit using the SAS GENMOD procedure by specifying the random error as Poisson distribution, and the link function, the identity link [27]. In addition, we specified the ‘NO INTERCEPT’ option in the procedure, as an intercept term is not required in the model [27]. The 95% confidence intervals (95% CI) for PAF and CPW estimates were derived by the bootstrap method (10,000 bootstrapping for each estimate) [23].

### Interpretation of the Causal-pie Weights

**Figure 1:**
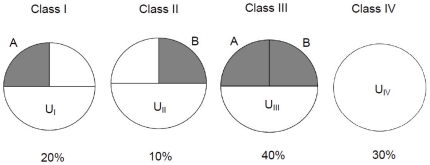
Causal pies and causal-pie weights for a hypothetical example. Four possible classes of causal pies for two hypothetical binary risk factors, A and B, in disease causation. The numbers shown below the pies are the causal-pie weights.

We use two hypothetical binary risk factors, A and B, for demonstration. There are four possible classes of causal pies (see figure 1): (I) the disease can occur if risk factor A and other unknown factors, U_I_, are present; (II) the disease can occur if risk factor B and other unknown factors, U_II_, are present; (III) the disease can occur if both risk factors A and B, as well as other unknown factors, U_III_, are present; and (IV) the disease can occur with neither A nor B being present, as long as some unknown factors, U_IV_, are present. In this example, class III causal pie is the most important. It has a CPW of 40%, implying that 40% of the patients had developed the disease contingent on risk factors A and B being simultaneously present (or stated differently, these patients developed the disease through interaction/synergism of the two risk factors). The CPW of class IV causal pie is 30%. This implies that 30% of the patients occurred through some certain pathways that involve none of risk factors A and B. A CPW=20% for class I implies that 20% of the patients had developed the disease due to the independent effect of risk factor A. A CPW=10% for class II implies that only 10% of the patients were the results of the independent effect of risk factor B.

## Results

Table 1 presents the baseline characteristics of the study participants (viral factors and habits of cigarette smoking and alcohol drinking) and the age-adjusted RRs of developing HCC. (Age-adjusted RRs were obtained from a multiplicative Poisson regression model including attained age as covariates: ‘30–39’, ‘40–49’, ‘50–59’, ‘60–69’, ‘70+’.) It can be seen that all factors except for smoking are significantly associated with HCC risk. A significant dose-response relation can be seen between viral load of HBV and HCC risk. Compared to individuals with negative HBsAg status, the RRs are 2.9 (HBV DNA level of <10^4^), 7.0 (HBV DNA between 10^4^ and 10^5^), 12.7 (HBV DNA between 10^5^ and 10^6^), and 22.6 (HBV DNA
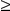
10^6^), respectively. HCV status is also associated with HCC risk. Those with detectable HCV RNA, irrespectively of the level being high (RR=6.1) or low (RR=6.1), have higher risk of HCC than those with undetectable HCV RNA (RR=1.6) and those with negative anti-HCV (RR=1.0). For individuals having ever drank, the risk of HCC is 1.4 fold higher than those never.

**Table 1:** Baseline characteristics and the age-adjusted rate ratios for developing hepatocellular carcinoma for a total of 11,801 men who were free of liver cirrhosis and hepatocellular carcinoma at study entry.

Baseline Characteristics	Total	Person-year	Number of Cases	Age-adjustedRate Ratio^a^	p-value^b^
**HBV status**					
HBsAg (—)	9458	141984.1	98	1.0	
HBV DNA 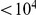 c	1250	18789.7	37	2.9	<0.01
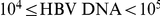 c	398	5887.9	29	7.0	<0.01
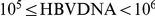 c	281	3961.0	37	12.7	<0.01
HBV DNA 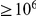 c	414	5544.6	97	22.6	<0.01
**HCV status**					
Anti-HCV (—)	11287	168883.1	242	1.0	
HCV RNA undetectable^d^	117	1743.6	4	1.6	0.17
HCV RNA detectable: Low^d^ **^, ^** e	198	2685.0	26	6.1	<0.01
HCV RNA detectable: High^d^ **^, ^** e	199	2855.5	26	6.1	<0.01
**Cigarette smoking**					
Never	5141	78375.3	122	1.0	
Ever	6660	97791.9	176	1.1	0.18
**Alcohol drinking**					
Never	9371	140886.5	217	1.0	
Ever	2430	35280.7	81	1.4	<0.01

a obtained from a multiplicative Poisson regression model including attained age as covariates: ‘30–39’, ‘40–49’, ‘50–59’, ‘60–69’, ‘70+’.

b one-sided p-value.

c also with HBsAg (+).

d also with anti-HCV (+); the detection limit is 25 IU/mL.

e the cut-off point is median RNA loads of study subjects with detectable quantity.

Table 2 presents the final additive Poisson regression model (after adjusting for age). All four incremental codes for the main effect of HBV status are significant and retained in the final model, including ‘HBsAg (+)’, ‘HBV DNA
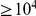
’, ‘HBV DNA
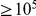
’ and ‘HBV DNA
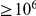
’. Of the total three incremental codes for the main effect of HCV RNA, only one is significant: ‘HCV RNA Detectable’. In addition, the final model contains two interaction terms, ‘HBsAg (+) × Alcohol Drinking’ and ‘HBV DNA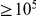 × Anti-HCV (+)’.

**Table 2:** The final additive Poisson model based on the data of a total of 11,801 men who were free of liver cirrhosis and hepatocellular carcinoma at study entry.

Variables^a^	Regression Coefficients( 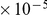 )	Standard Errors( 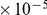 )	p-value^b^
Intercept	7.7	0.9	<0.01
HBsAg (+)	83.1	30.0	<0.01
HBV DNA 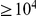 c	299.1	94.2	<0.01
HBV DNA 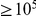 c	394.7	173.3	0.01
HBV DNA 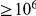 c	810.3	230.0	<0.01
HCV RNA Detectable^d^	777.0	126.1	<0.01
HBsAg (+) ×Alcohol Drinking	193.2	92.9	0.02
HBV DNA 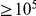 c×Anti-HCV (+)	2273.1	1325.0	0.04

a dummy code for alcohol drinking; incremental codes for HBV and HCV status.

b one-sided p-value.

c also with HBsAg (+).

d also with anti-HCV (+).

Table S2 presents the observed and the expected number of newly-developed HCC patients (and also the crude and the model-based HCC incidence rates), based on the final model in Table 2. [Table S2 has a total of 30 ‘cells’: 2 (for alcohol) ×3 (for HCV) ×5 (for HBV). This is based on the cut-offs used in the final model in Table 2.] It can be seen that the observed and the expected are in good agreement (goodness-of-fit p-values are 0.68 when the data is grouped into a total of ten cells, and 0.14, when grouped into a total of four cells).

**Figure 2:**
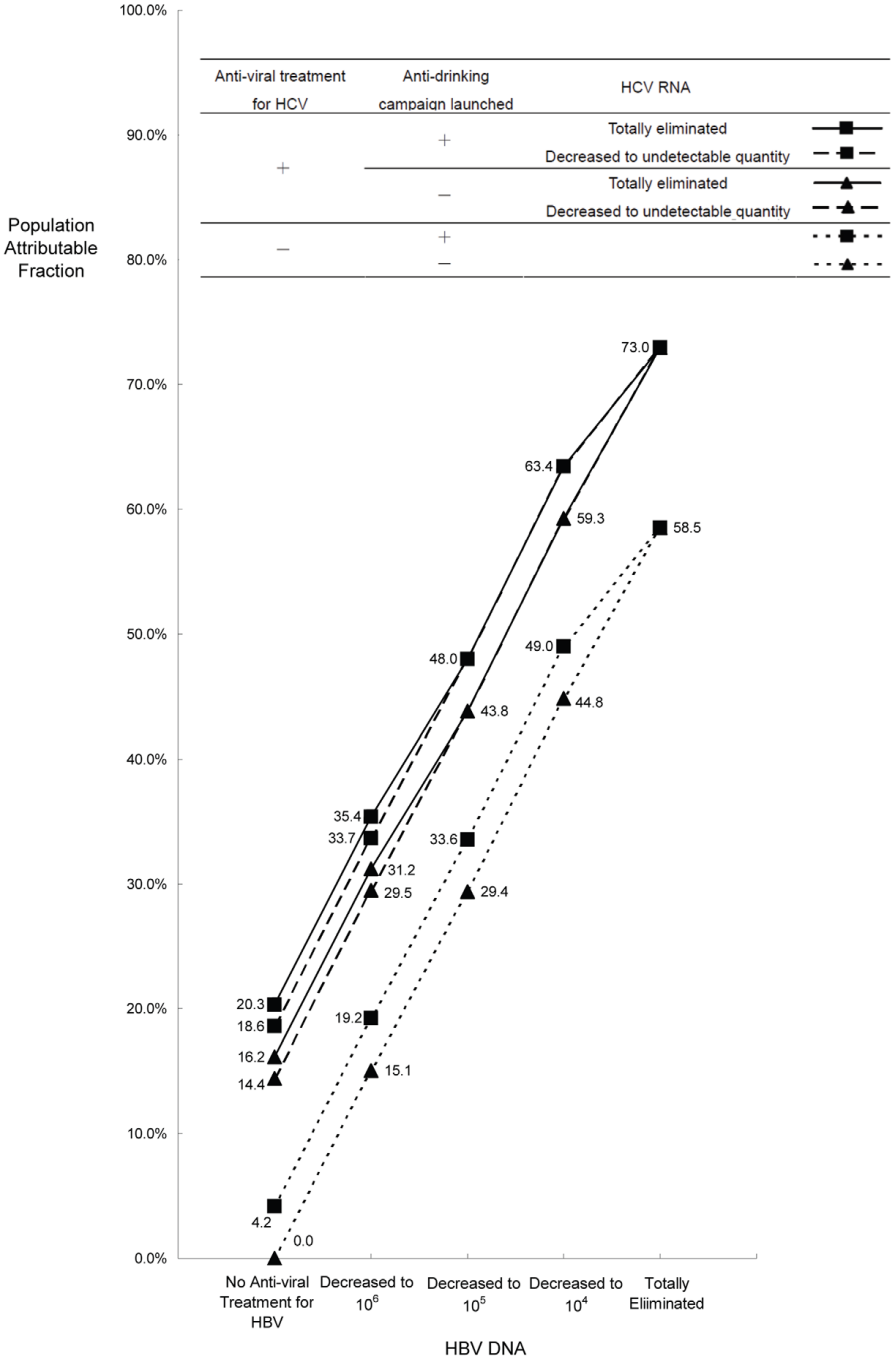
Population attributable fractions under various intervention strategies.

The PAFs based on the final additive Poisson regression model are presented in figure 2, from which the magnitudes of case-load reduction under various public health intervention or treatment strategies can be inferred. It can be seen that the proportion of HCC case reduction increases as HBV DNA level is being lowered down. The increments are rather striking. We observe a chance to reduce around 60% HCC risk moving from no HBV-related intervention to the total elimination of virus. An additional ∼15% (or ∼5%) reduction can be expected, if the HBV-related intervention is coupled with an HCV-related intervention (or an anti-drinking campaign). It makes little difference, though, whether HCV RNA is totally eliminated or is decreased to undetectable quantity.

**Figure 3:**
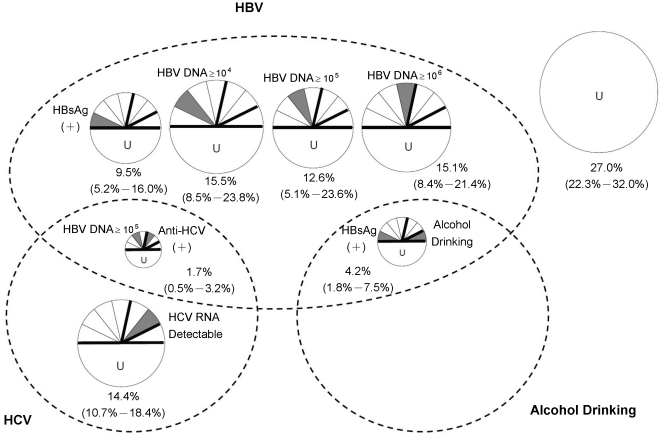
Causal-pie weights and the bootstrapped 95% confidence intervals for newly-developed hepatocellular carcinoma. A total of eight classes of causal pies (U: unmeasured factors) are presented. The eight pies are grouped into three intersecting sets (the dotted circles).

Figure 3 presents the CPWs (and the 95% CIs) of the classes of causal pies for HCC. (Note that we did not distinguish the different unknown factors in different classes of causal pies. Rather, we simply used a capital U to acknowledge them.) There are a total of eight classes of causal pies (eight ‘pies’ in the figure) playing important roles in HCC development and each of them with weights significantly larger than zero. We let the areas of the pies to be in proportional to their respective CPWs. A CPW for a particular class of causal pies represents the proportion of HCC cases attributable to that class. For example, the CPW for the class of causal pies that contain HBsAg (+) as one of its component causes is 9.5%. This means that 9.5% of HCC cases had developed the disease because of their being HBV carriers regardless of their HBV DNA levels. (Note that an HBV carrier who had developed HCC may not necessarily have acquired the disease because of his/her carrier status. He/she may well have developed the disease through other class of causal pies, if he/she had completed all the component causes of that class.) As another example, the CPW for the class of causal pies that includes HBV DNA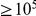 and Anti-HCV (+) is 1.7%. This means that 1.7% of HCC cases occurred because of the synergistic effect of HBV DNA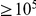 and Anti-HCV (+).

For clarity, we group the eight classes of causal pies using the Venn diagram (the three dotted circles in Figure 3, representing the three sets of ‘HBV’, ‘HCV’ and ‘alcohol drinking’, as well as their intersections). The HBV ‘circle’ encompasses a total of four dose-response classes of causal pies representing the independent HBV effect (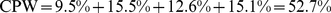), one class of HBV-alcohol interaction with small weight (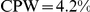), and one class of HBV-HCV interaction with even smaller weight (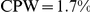). All told, the HBV circle has a total apportioned weight of 55.7%. (All the independent HBV effect contributes to the HBV circle, while each of the HBV-alcohol and HBV-HCV interaction classes contributes only half of its weight to the HBV circle: 55.7%=52.7%×100%+1.7%×50%+4.2%×50%.) The HCV circle encompasses an HCV-only class and an HBV-HCV interaction class with a total apportioned weight of 14.4%×100%+1.7%×50%=15.3%. The alcohol drinking circle is the smallest. It encompasses only one class of causal pies (the HBV-alcohol interaction class), which contributes half of its weight to this circle (apportioned weight
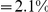
=4.2%×50%). Outside the circles, the ‘all-U’ class of causal pies (the class of causal pies composed exclusively of unknown or unmeasured factors) accounts for the remaining weight of 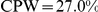.

To gauge the impacts of selection bias, we re-analyzed the data a number of times, by excluding (1) men residing in a specific township one at a time; (2) men in a particular age group one at a time; and (3) men with low-level education, the results were essentially the same (table S3). We also performed the analysis excluding patients who were diagnosed as HCC within one year after enrollment. The results were also essentially the same (table S3).

## Discussion

Due to technological or cost constraint, the detection of HBV and HCV infection had been largely limited to using serological tests (for HBsAg and anti-HCV, respectively) in earlier epidemiologic studies on HCC [3]. In this study however, we not only can detect HBV and HCV infection but can also directly measure the quantities of the viruses in infected persons (the viral loads). Therefore, we are able to examine the dose-response relation between viral loads and HCC risk in greater details. For HBV, we found that the higher its DNA level, the higher the risk of HCC. As for HCV, the risk for HCC stays pretty much the same as long as its RNA level is detectable irrespectively of how high it is. Viral pathogenesis may explain the observed disparity in the dose-response relations between the two viruses. HBV is a DNA virus and involves tumorgenesis process by its direct integration into the host genome [10]. If a person has a high HBV DNA level, the virus is more likely to be integrated into the host genome. The risk of HCC would therefore be higher as HBV DNA level increases. The HCV, being an RNA virus, involves a different mechanism to cause HCC, however. If the amount of HCV virus was sufficiently detected by the host immune system, it would take defense to protect human body. But more or less, liver injury would be resulted [28], [29]. Therefore, the risk of HCC is expected to be higher once the amount of HCV is accumulated to a certain level. And over the threshold, since the immune response was induced, there would be no difference on HCC risk between individuals who are with lower or higher HCV virus level.

It is of interest to find that the interaction between HBV and HCV on the risk of HCC is also dose-dependent—the HBV-HCV interaction occurs only when HBV DNA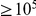. Previous *in vitro* studies showed that co-infection of HBV and HCV leads to a mutual suppression between the two viruses [30]. We postulate that the suppression of the HCV on the HBV is less effectual once the viral load of the latter is becoming too high (HBV DNA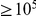). With this threshold crossed, the full potentials of the co-infection of the two viruses can be unleashed and we then observe a significant viral interaction on HCC risk.

In this study, we found an interaction effect between alcohol drinking and positivity of HBsAg on HCC risk, but alcohol drinking itself lacks an independent effect. Previous studies conducted in Western countries have shown an increased risk of HCC for those who consumed alcohol for more than 80 grams per day (the independent effect of alcohol drinking) [22]. By comparison, the average dose of alcohol consumption and the prevalence of alcoholics in Asia are much lower than those in the Western countries [22], [31]. In Taiwan, individuals with problematic alcohol consumption are also proved to be more prevalent in the aboriginal group than in other ethnic group [32]. This may explain why this study (mainly focusing on non-aboriginal Fukienese and Hakka) did not show a significant independent effect of alcohol drinking. We however caution that even a relatively mild drinking as in this study suffices to increase the risk for HCC—not for everyone though, but at least for those HBV carriers.

Epidemiologists are accustomed to characterize the association between a risk factor and a disease using an RR index and to demonstrate a risk factor's contribution to the disease burden by using a PAF index. In addition to these commonly used indices, in this study we further use the CPW index to represent the relative importance of the various classes of causal pies for HCC. We also draw a causal Venn diagram. The diagram groups the various classes of causal pies into three circles (HBV, HCV and alcohol drinking, respectively) and their intersections. From this, an overall picture of HCC causation then emerges. The big players are HBV (total apportioned weight: 55.7%) and HCV (15.3%), whereas alcohol drinking plays a much lesser role (2.1%).

Outside the Venn diagram, there is an all-U class of causal pies containing none of the measured factors in this study as its component causes. It accounts for the remaining weight (27.0%). (This weight estimate is rather stable, judging from its bootstrapped confidence interval. Moreover, we re-run our analysis using different 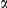 levels. The weight estimates also appear to be similar: 27.1% when 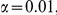 and 26.9% when 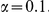) An all-U weight as large as 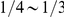 suggests that there are still many risk factors of HCC awaiting discovery, other than the HBV, HCV and alcohol drinking that are measured and analyzed in this study.

## Supporting Information

Table S1 **Distributions of demographic data among male responders and non-responders.**
(DOC)

Table S2 **The observed/expected number of patients and the crude/model-based rates based on the final additive Poisson model.**
(DOC)

Table S3 **Causal-pie weights (%) for a total of eight classes of causal pies (U: unmeasured factors) by a series of exclusion criteria.**
(DOC)
