# Supplementary material for: Fifteen-Year Population Attributable Fractions and Causal Pies of Risk Factors for Newly Developed Hepatocellular Carcinomas in 11,801 Men in Taiwan
Source: PLoS One. 2012 Apr 10;7(4):e34779. doi: 10.1371/journal.pone.0034779 (PMC3323561; doi:10.1371/journal.pone.0034779)
Supplement: Table S1 — Distributions of demographic data among male responders and non-responders. (DOC) [file pone.0034779.s001.doc]

TABLE S1. Distributions of demographic data among male responders and non-responders.

|  | % | |
| --- | --- | --- |
|  | Non-responder  N=35102 | Responder  N=11972 |
| Residential Area |  |  |
| Sanchi | 7.9 | 6.3 |
| Paihsa | 3.8 | 4.8 |
| Potzu | 19.6 | 15.7 |
| Chutung | 29.6 | 20.4 |
| Makung | 20.9 | 27.5 |
| Kaoshu | 13.5 | 18.7 |
| Huhsi | 4.7 | 6.7 |
| Age |  |  |
| 30－34 | 21.9 | 13.1 |
| 35－39 | 19.6 | 15.5 |
| 40－44 | 14.6 | 13.0 |
| 45－49 | 10.6 | 11.2 |
| 50－54 | 12.1 | 15.3 |
| 55－59 | 11.6 | 16.1 |
| 60－64 | 9.6 | 15.8 |
| Education* |  |  |
| Low-level | 2.4 | 9.1 |
| High-level | 97.6 | 90.9 |

* Low-level education: uneducated or primary-school educated.

High-level education: junior high school, senior high school, vocational school or college/graduate school.
