# Supplementary material for: Fifteen-Year Population Attributable Fractions and Causal Pies of Risk Factors for Newly Developed Hepatocellular Carcinomas in 11,801 Men in Taiwan
Source: PLoS One. 2012 Apr 10;7(4):e34779. doi: 10.1371/journal.pone.0034779 (PMC3323561; doi:10.1371/journal.pone.0034779)
Supplement: Table S2 — The observed/expected number of patients and the crude/model-based rates based on the final additive Poisson model. (DOC) [file pone.0034779.s002.doc]

TABLE S2. The observed/expected number of patients and the crude/model-based rates based on the final additive Poisson model.

|  | Total | Person-Year | Number of Patients  Observed / Expected | Incidence Rates ($\times10^{-5}$)  Crude / Model-based |
| --- | --- | --- | --- | --- |
| Alcohol Drinking: Never |  |  |  |  |
| Anti-HCV (－) |  |  |  |  |
| HBsAg (－) | 7139 | 108070.8 | 40 / 50.7 | 37.0 / 46.9 |
| HBV DNA$<10^{4}$ **^a^** | 954 | 14444.7 | 21 / 17.9 | 145.4 / 124.1 |
| $\text{ }10^{4}\leq\text{HBV DNA}<10^{5}$ **a** | 317 | 4724.4 | 22 / 20.0 | 465.7 / 423.2 |
| $\text{ }10^{5}\leq\text{HBV DNA}<10^{6}$ **a** | 223 | 3162.0 | 27 / 25.7 | 853.9 / 812.4 |
| $\text{HBV DNA}\geq10^{6}$ **a** | 312 | 4291.0 | 65 / 69.3 | 1514.8 / 1616.1 |
| HCV RNA Undetectable **^b^** |  |  |  |  |
| HBsAg (－) | 77 | 1166.2 | 0 / 0.65 | 0.0 / 55.3 |
| HBV DNA$<10^{4}$ **^a^** | 13 | 194.0 | 0 / 0.23 | 0.0 / 119.0 |
| $\text{ }10^{4}\leq\text{HBV DNA}<10^{5}$ **a** | 2 | 32.3 | 0 / 0.14 | 0.0 / 429.5 |
| $\text{ }10^{5}\leq\text{HBV DNA}<10^{6}$ **a** | 4 | 58.9 | 1 / 1.82 | 1697.0 / 3094.8 |
| $\text{HBV DNA}\geq10^{6}$ **a** | 4 | 46.5 | 2 / 1.81 | 4303.5 / 3889.2 |
| HCV RNA Detectable **^b^** |  |  |  |  |
| HBsAg (－) | 272 | 3852.3 | 31 / 32.0 | 804.7 / 830.7 |
| HBV DNA$<10^{4}$ **^a^** | 42 | 629.4 | 4 / 5.8 | 635.5 / 914.6 |
| $\text{ }10^{4}\leq\text{HBV DNA}<10^{5}$ **a** | 6 | 74.5 | 1 / 0.9 | 1342.2 / 1198.7 |
| $\text{ }10^{5}\leq\text{HBV DNA}<10^{6}$ **a** | 1 | 15.9 | 1 / 0.6 | 6274.7 / 3894.9 |
| $\text{HBV DNA}\geq10^{6}$ **a** | 5 | 64.7 | 2 / 3.0 | 3089.8 / 4695.7 |
| Alcohol Drinking: Ever |  |  |  |  |
| Anti-HCV (－) |  |  |  |  |
| HBsAg (－) | 1895 | 27888.6 | 17 / 13.4 | 61.0 / 48.0 |
| HBV DNA$<10^{4}$ **^a^** | 232 | 3369.3 | 11 / 10.7 | 326.5 / 316.2 |
| $\text{ }10^{4}\leq\text{HBV DNA}<10^{5}$ **a** | 72 | 1038.2 | 6 / 6.4 | 577.9 / 616.8 |
| $\text{ }10^{5}\leq\text{HBV DNA}<10^{6}$ **a** | 51 | 702.6 | 6 / 7.1 | 853.9 / 1005.9 |
| $\text{HBV DNA}\geq10^{6}$ **a** | 92 | 1127.5 | 27 / 20.4 | 2394.6 / 1811.7 |
| HCV RNA Undetectable **^b^** |  |  |  |  |
| HBsAg (－) | 13 | 185.7 | 0 / 0.1 | 0.0 / 58.2 |
| HBV DNA$<10^{4}$ **^a^** | 3 | 47.0 | 0 / 0.1 | 0.0 / 313.4 |
| $\text{ }10^{4}\leq\text{HBV DNA}<10^{5}$ **a** | 0 | 0.0 | 0 / 0.0 | NA / 575.4 |
| $\text{ }10^{5}\leq\text{HBV DNA}<10^{6}$ **a** | 1 | 12.2 | 1 / 0.4 | 8163.7 / 3257.8 |
| $\text{HBV DNA}\geq10^{6}$ **a** | 0 | 0.0 | 0 / 0.0 | NA / 4053.6 |
| HCV RNA Detectable **^b^** |  |  |  |  |
| HBsAg (－) | 62 | 761.3 | 10 / 6.3 | 1313.6 / 825.2 |
| HBV DNA$<10^{4}$ **^a^** | 6 | 97.3 | 1 / 1.1 | 1027.5 / 1113.3 |
| $\text{ }10^{4}\leq\text{HBV DNA}<10^{5}$ **a** | 1 | 16.0 | 0 / 0.2 | 0.0 / 1430.2 |
| $\text{ }10^{5}\leq\text{HBV DNA}<10^{6}$ **a** | 1 | 14.1 | 1 / 0.6 | 7103.9 / 4094.2 |
| $\text{HBV DNA}\geq10^{6}$ **a** | 1 | 12.6 | 1 / 0.6 | 7909.0 / 4853.5 |

**^a^** also with HBsAg (+)

**^b^** also with anti-HCV (+)
