# Supplementary material for: Fifteen-Year Population Attributable Fractions and Causal Pies of Risk Factors for Newly Developed Hepatocellular Carcinomas in 11,801 Men in Taiwan
Source: PLoS One. 2012 Apr 10;7(4):e34779. doi: 10.1371/journal.pone.0034779 (PMC3323561; doi:10.1371/journal.pone.0034779)
Supplement: Table S3 — Causal-pie weights (%) for a total of eight classes of causal pies (U: unmeasured factors) by a series of exclusion criteria. (DOC) [file pone.0034779.s003.doc]

TABLE S3. Causal-pie weights (%) for a total of eight classes of causal pies (U: unmeasured factors) by a series of exclusion criteria.

|  | Classes of Causal Pies | | | | | | | |
| --- | --- | --- | --- | --- | --- | --- | --- | --- |
|  | All-U | HBsAg (＋) | HBV DNA $\geq10^{4}$ | HBV DNA $\geq10^{5}$ | HBV DNA $\geq10^{6}$ | HCV RNA Detectable | HBsAg (＋) × Alcohol Drinking | HBV DNA$\geq10^{5}$ × Anti-HCV (＋) |
| Original data | | | | | | | | |
| Mean | 27.0 | 9.5 | 15.5 | 12.6 | 15.1 | 14.4 | 4.2 | 1.7 |
| (95%CI) | (22.3－32.0) | (5.2－16.0) | (8.5－23.8) | (5.1－23.6) | (8.4－21.4) | (10.7－18.4) | (1.8－7.5) | (0.5－3.2) |
| Excluding men residing in | | | | | | | | |
| Huhsi | 26.1 | 9.9 | 15.5 | 12.4 | 14.8 | 15.1 | 4.5 | 1.6 |
| Chutung | 25.6 | 11.4 | 14.6 | 12.5 | 15.3 | 14.2 | 4.4 | 1.9 |
| Potzu | 25.6 | 10.3 | 15.5 | 17.5 | 14.0 | 11.5 | 3.3 | 1.5 |
| Makung | 26.3 | 6.4 | 15.3 | 12.7 | 13.1 | 18.3 | 5.7 | 2.2 |
| Paihsa | 28.8 | 8.7 | 18.1 | 13.4 | 13.0 | 11.9 | 4.9 | 1.3 |
| Kaoshu | 28.2 | 9.2 | 15.8 | 8.3 | 17.4 | 16.4 | 2.7 | 2.0 |
| Sanchi | 27.6 | 10.1 | 13.9 | 12.4 | 16.6 | 14.1 | 3.8 | 1.6 |
| Excluding men aged | | | | | | | | |
| 30－34 | 25.3 | 11.0 | 14.8 | 11.3 | 17.4 | 14.9 | 3.7 | 1.7 |
| 35－39 | 27.0 | 10.0 | 14.5 | 11.3 | 15.2 | 16.6 | 3.9 | 1.5 |
| 40－44 | 27.6 | 10.4 | 15.9 | 14.5 | 11.3 | 14.7 | 3.9 | 1.7 |
| 45－49 | 29.7 | 7.7 | 14.8 | 12.8 | 14.2 | 15.3 | 3.8 | 1.7 |
| 50－54 | 27.4 | 8.1 | 16.5 | 11.5 | 15.2 | 12.9 | 6.4 | 2.0 |
| 55－59 | 29.8 | 8.0 | 18.0 | 10.3 | 13.1 | 14.6 | 4.6 | 1.6 |
| 60－64 | 22.2 | 11.4 | 14.2 | 16.3 | 18.3 | 12.9 | 3.1 | 1.8 |
| 65+ | 26.6 | 9.6 | 15.0 | 13.1 | 15.2 | 14.6 | 4.3 | 1.7 |
| Excluding men with  low-level education^a^ | 26.7 | 8.8 | 13.2 | 15.9 | 14.0 | 14.8 | 5.0 | 1.8 |
| Excluding HCC prevalent  cases^b^ | 26.2 | 7.7 | 14.2 | 13.4 | 16.3 | 15.4 | 5.0 | 1.9 |

^a^ Low-level education: uneducated or primary-school educated.

^b^ HCC prevalent cases: patients who were diagnosed as HCC within one year after enrollment.
